# Intraoperative ketamine for reduction in postpartum depressive symptoms after cesarean delivery: A double‐blind, randomized clinical trial

**DOI:** 10.1002/brb3.1715

**Published:** 2020-08-18

**Authors:** Jiaxin Yao, Tingting Song, Yue Zhang, Nan Guo, Ping Zhao

**Affiliations:** ^1^ The Second Department of Anesthesia Shengjing Hospital of China Medical University Shenyang China

**Keywords:** cesarean, depression, ketamine, pain, postpartum

## Abstract

**Background:**

Postpartum depression (PPD) is a common mental disease happens in perinatal period. Ketamine as an anesthesia and analgesia drug has been used for a long time. In recent years, ketamine is proved to have an antidepression effect with a single administration. We hypothesized that intraoperative ketamine can reduce postpartum depressive symptoms after cesarean delivery.

**Methods:**

In a randomized, double‐blind, placebo‐controlled study trail, healthy women scheduled for cesarean delivery were randomly assigned to receive intravenous ketamine (0.25 mg/kg diluted to 5 ml with 0.9% saline) or placebo (5 ml of 0.9% saline) within 5 min following clamping of the neonatal umbilical cord. The primary outcome was the degree of postpartum depressive symptoms, which was evaluated by Edinburgh Postnatal Depression Scale (EPDS, a threshold of 9/10 was used) at 1 week, 2 weeks, and 1 month after delivery. The secondary outcome was the numerical rating scale (NRS) score of pain at 2 days postpartum. This trail is registered in the Chinese Clinical Trial Registry, number ChiCTR1900022464.

**Results:**

Between 26 January 2019 and 15 July 2019, 502 subjects were screened and 330 were randomly allocated: 165 (50%) to the ketamine group and 165 (50%) to the placebo group. There were significant differences in the degree of postpartum depressive symptoms between subjects in the ketamine group and the placebo group at 1 week postpartum (13.1% vs. 22.6%, respectively; *p* = .029). However, no difference was found between subjects in the two groups at 2 weeks (11.8% vs. 16.8%, respectively; *p* = .209) and 1 month postpartum (10.5% vs. 14.2%, respectively; *p* = .319). The NRS score of wound pain (3.0 ± 0.9 vs. 4.0 ± 1.0, respectively; *p* < .001) and uterine contraction pain (3.0 ± 0.9 vs. 4.1 ± 0.9, respectively; *p* < .001) was lower in the ketamine group at 2 days postpartum compared with placebo group. The prevalence of headache, hallucination, and dizziness was higher in the ketamine group than the placebo group during the operation.

**Conclusions:**

Operative intravenous ketamine (0.25 mg/kg) can reduce the postpartum depressive symptoms for 1 week. The long‐time effect is remained to be seen.

## Graphical abstract

Operative intravenous ketamine (0.25 mg/kg) can prevent postpartum depressive symptoms for 1 week. The long‐time effect is remained to be seen.

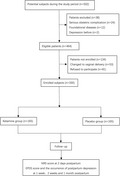

## 1 INTRODUCTION

Postpartum depression (PPD) is a major depressive episode “with peripartum onset of mood symptoms occurring during pregnancy or within 4 weeks following delivery” included in the American Psychiatric Association's Diagnostic and Statistical Manual of Mental Disorders, fifth edition (DSM‐5). Symptoms of postpartum depression often include sleep disturbance, anxiety, irritability, a feeling of being overwhelmed, and an obsessional preoccupation with the baby's health and feeding. What's more, 70% of new mothers have mild depressive symptoms called “baby blues,” which peak between 2 and 5 days after delivery and typically, include weepiness, sadness, mood lability, irritability, and anxiety. “Blues” do not seriously impair functioning or include psychotic symptoms; typically, these symptoms begin to abate spontaneously within 2 weeks, although some cases will progress to postpartum depression (Wisner, Parry, & Piontek, 2002). PPD can have a negative impact on family harmony (Ngai & Ngu, 2015), couple relationship, maternal health, and infant health and development (Paulson, Dauber, & Leiferman, 2006).

The estimated prevalence of postpartum depression ranges from 6.5% to 12.9% or even higher in lower‐income and middle‐income countries (Gaynes et al., 2005; Munk‐Olsen, Laursen, Pedersen, Mors, & Mortensen, 2006). Before the 1980s, PPD was seldom reported in China. In recent years, the reported rates of PPD from studies enrolling Chinese populations have ranged from 10% to 20% (Hansotte, Payne, & Babich, 2017; Meltzer‐Brody et al., 2018; Xie et al., 2007). Many factors are found to increase the likelihood of getting PPD, included cesarean delivery (Xu, Ding, Ma, Xin, & Zhang, 2017; Yang, Shen, Ping, Wang, & Chien, 2011). Cesarean might induce adverse physiological outcomes, such as infection, postpartum hemorrhage, injury to the ureter and bladder, uterine rupture, chronic pelvic pain, and gastrointestinal dysfunction (Murphy, Liebling, Verity, Swingler, & Patel, 2001). These adverse outcomes and surgical trauma might enhance stress, which might affect the psychological function and increase the risk of PPD for mothers (Desborough, 2000). Compared with women who delivered vaginally, women who had a cesarean delivery are more likely to have PPD.

Ketamine, an N‐methyl‐D‐aspartate (NMDA) antagonist, has been used as an anesthesia drug for more than half a century. When used in subanesthetic doses, has analgesic properties that have been used for treatment of acute and chronic pain. Two meta‐analyses revealed that low‐dose ketamine administrated during the perioperative period can reduce postoperative pain intensity (Brinck et al., 2018) and prevent chronic pain (Chaparro, Smith, Moore, Wiffen, & Gilron, 2013), which may be related to reduction in central sensitization and hyperalgesia via inhibition of NMDA receptors. Over the past decade, it has been provoked a single administration of ketamine elicits fast (in as little as half an hour) and sustained antidepressant effects both in human (Berman et al., 2000; Zarate et al., 2006)and animal models of depression. There are some potential mechanisms of antidepressant actions of ketamine. MK‐801, a noncompetitive NMDA receptor antagonist, produced antidepressant‐like actions in the animal model of depression (Trullas & Skolnick, 1990). Ketamine can also increase hippocampal brain‐derived neurotrophic factor levels, which may be important for producing a rapid onset of antidepressant action (Garcia et al., 2008) A recent study found ketamine could quickly elevate mood by blocking NMDAR receptor‐dependent bursting activity of the lateral habenula neurons to disinhibit downstream monoaminergic reward centers and provide a framework for developing new rapid‐acting antidepressants (Yang et al., 2018).

Accordingly, we hypothesized that intraoperative ketamine can reduce the postpartum depressive symptoms after cesarean delivery.

## 2 METHODS

### 2.1 Study design

In this double‐blind, randomized prospective trial, we explored the severity of postpartum depressive symptoms in pregnant woman who use ketamine or not any intravenous analgesic during cesarean delivery. The study protocol was approved by the Shengjing Hospital of China Medical University, Shenyang, Liaoning, and written informed consent was obtained from all subjects. The study was registered in the Chinese Clinical Trial Registry, number ChiCTR1900022464.

The inpatient of obstetrics from 26 June 2019 to 15 July 2019 in Shengjing hospital was enrolled. Demographic and basic information were collected for all patients, including age, body mass index, employment, partner employment, number of births, gestational age, and obstetric complications. Eligibility criteria included the following: (a) undergoing cesarean delivery in Shengjing hospital, (b) the age of the pregnant is ≥20 and ≤40, (c) the birth age is ≥37 and ≤42 weeks, and (d）the birth weight is ≥2,500 and ≤4,000 g. Exclusion criteria included the following: (a) The pregnant has experienced depression and has been diagnosed as depression by a psychiatrist before, (b) the pregnant has experienced domestic violence before, (c) the pregnant with a serious obstetric complication or serious foundational diseases, (d) the new born has serious genetic or congenital diseases, (e) multiple pregnancy, and (f) the maternal weight is <50 or >100 kg, and the maternal height is <150 or >180 cm.

### 2.2 Randomization and masking

Randomization assignments were from a computer‐generated random number table. The group assignment and the investigational drug protocol were concealed in sequentially numbered, sealed, opaque envelopes. On the day of caesarean delivery, the assistant, who was not blinded to the study protocol opened the envelopes in the order of subject enrolment, reconstituted the investigational drug into a 5‐mL syringe and then labeled the syringe with the assigned subject ID. The subjects, anesthesiologists, and follow‐up investigators were all blinded to the subjects’ group assignments. This information was concealed until the subject recruitment and follow‐up had been completed.

### 2.3 Procedures

All patients received a spinal anesthesia at the level of L3–L4 or L2‐L3. The spinal block solution was bupivacaine 7.5 mg (1.5 ml 0.5% bupivacaine in saline) given at 1 ml/5 s to achieve a block level up to T4–T6. The operating table was tilted 30°to the left when the pregnant turned to a supine position. The patient's vital signs were closely monitored, and phenylephrine (iv) was administered when necessary. Within 5 min after clamping the neonatal umbilical cord, ketamine (0.25 mg/kg diluted to 5 ml with 0.9% saline) or placebo (5 ml of 0.9% saline) was slowly injected (iv) by the anesthesiologist who was blinded to the study protocol. The prepregnant weights of the subjects were recorded for the dose calculation. Five and fifteen minutes after the administration of the study drugs, and before the patient leaving the operation room, the anesthesiologist asked the subject if she had headache, vomiting, dizziness, or hallucination. At the end of surgery, epidural injection of 0.5 mg/ml morphine 4 ml was used for postoperative analgesia for each subject.

### 2.4 Outcome measures

The primary outcome was the degree of postpartum depressive symptoms which is assessed by the Edinburgh Postnatal Depression Scale (EPDS) score at 1 week, 2 weeks, and 1 month postpartum assessed by phone. EPDS is a questionnaire which is recommended by both the American College of Obstetricians and Gynecologists (Sit et al., 2015) and the American Academy of Pediatrics (Committee on Psychosocial Aspects of Child & Family Health, 2001) as a method of identifying possible postpartum depression. As a screening questionnaire for PPD, the positive findings from it should lead to a comprehensive clinical interview to ascertain a diagnosis (Antenatal & Postnatal Mental Health, 2014). It has been translated into Chinese assessing Chinese patients. When the threshold of 9/10 is used as the diagnostic criterion, the sensitivity and specificity are 82% and 86%, respectively (Lee et al., 1998), which can reduce the failed detection of cases than the threshold score of 12/13. The threshold of 9/10 may be appreciate if the scale is considered for routine use by primary care workers (Cox, Holden, & Sagovsky, 1987). Here, we used an EPDS score >9 as the cutoff value to screen postpartum depression. We defined the subjects whose EPDS scores were >9 had severer depressive symptoms and higher risk of PPD, whom we recommended to go to the psychiatric clinic to ascertain diagnoses. The secondary outcome was the pain intensity, which was assessed by study personnel using numerical rating scale (NRS) pain scores at 2 days postpartum. We also measured the sleep duration at 2 days postpartum.

### 2.5 Statistical analysis

The prevalence of PPD varied from country to country; in lower‐income and middle‐income countries, it can be higher (Howard et al., 2014). In China, the estimated prevalence of PPD is 22%. What is more, cesarean section delivery as an independent risk factor can increase the risk of PPD (Xu, Ding, et al., 2017). We aimed that the incidence rate of postpartum depression can be reduced from 20% to 10% by the use of ketamine. Group sample size of 150 achieved 80% power to detect this difference. The significance level of the test was targeted at 0.05. In order to account for dropouts, the study randomized 165 patients to each group. The primary outcome measure, the degree of postpartum depressive symptoms which was evaluated by the EPDS, was compared between groups using the chi‐square test. The score of EPDS was performed by analysis of variance (AVONA). The NRS pain score was compared between groups using the Mann–Whitney *U* test. A *p* value <.05 was required to reject the null hypothesis.

## 3 RESULT

**FIGURE 1:**
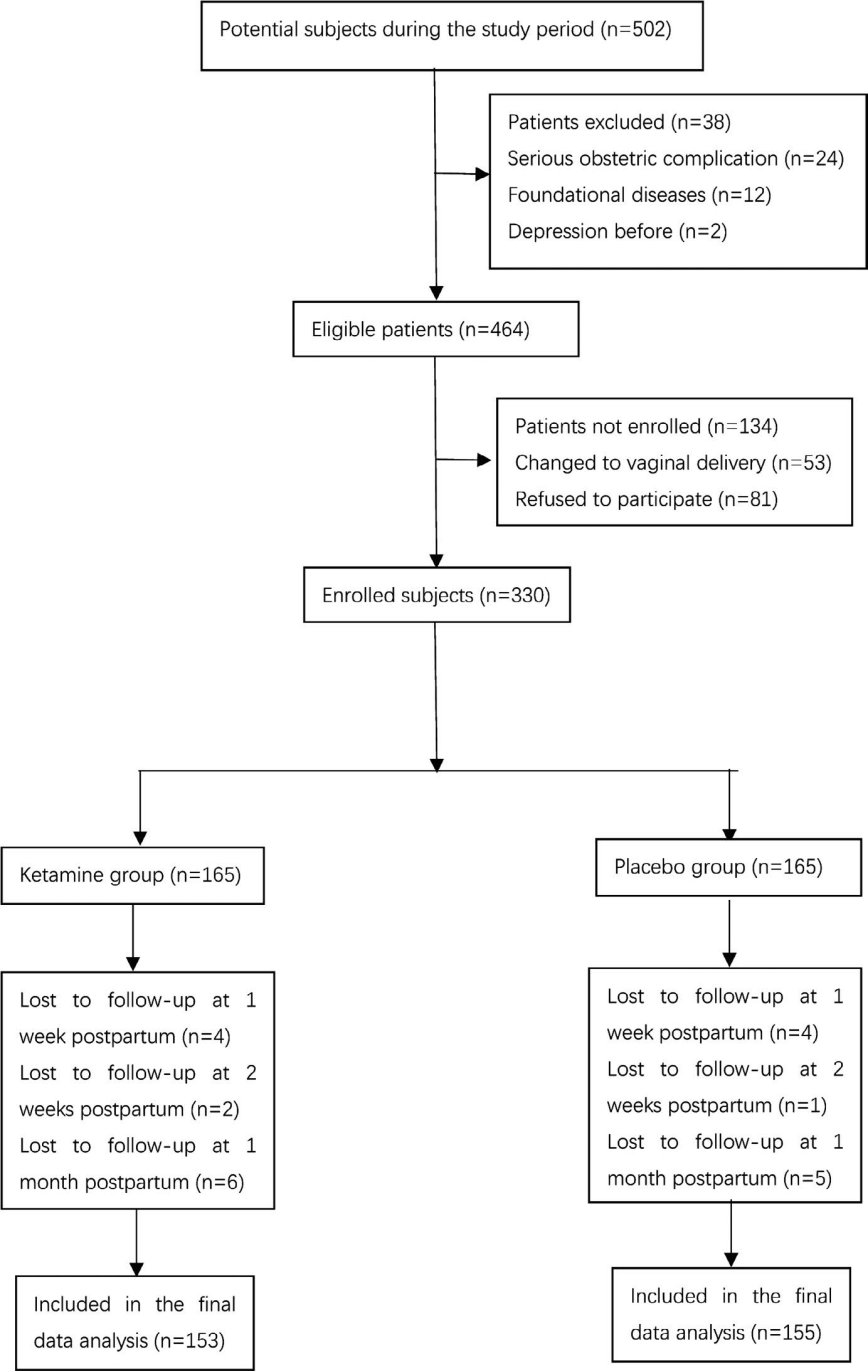
Patient flow

**FIGURE 2:**
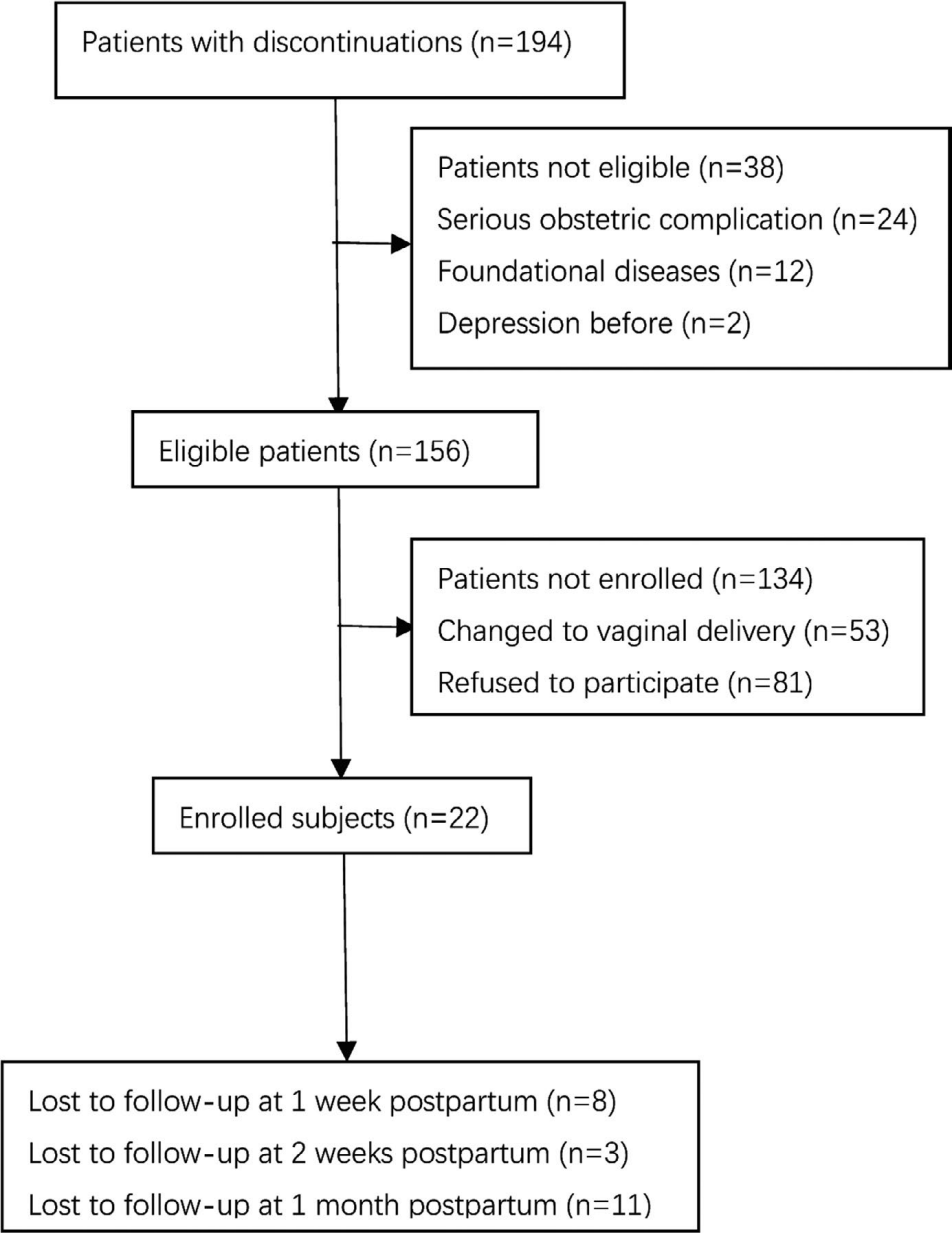
Patient flow with discontinuation

Between 26 June 2019 and 15 July 2019, 502 subjects were screened and 330 were randomly allocated: 165 (50%) to the ketamine group and 165 (50%) to the placebo group. 12 subject were lost to follow‐up in the ketamine group, and 10 subjects were lost to follow‐up in the placebo group. In the end, data from 153 subjects in the ketamine group and 155 in the placebo group were included in the analysis (Figure 1). The total number of patients with discontinuations was 194 (Figure 2). Subjects’ characteristics or the intraoperative maternal and neonatal general parameters (Table 1) did not differ between groups.

**TABLE 1:** Baseline characteristics of subjects

	Ketamine (*n* = 153)	Saline (*n* = 155)	*p*
Age (years)	30 ± 4	30 ± 3	.749
Body mass index (kg/m^2^)	29 ± 3	28 ± 3	.346
Employment	121 (79.1%)	113 (72.9%)	.284
Partner employment	141 (92.2%)	147 (94.8%)	.365
Fetus			
First‐born	111 (72.6%)	117 (75.5%)	.557
Second‐born	42 (27.4%)	38 (24.5%)	.557
Gestational age (day)	270 ± 9	269 ± 11	.525
Pregnancy with obstetric disease	41 (26.5%)	45 (29%)	.662
Neonatal gender			
Male	67 (43.8%)	82 (52.9%)	.110
Female	86 (56.2%)	73 (47.1%)	.110
Neonatal body weight (g)	3,312 ± 433	3,371 ± 329	.179

For the primary outcome of our study, there were significant differences in the degree of postpartum depressive symptoms between the ketamine group and the placebo group at 1 week postpartum (13.1% vs. 22.6%, respectively; *p* = .029). However, no significant differences were found between two groups at 2 weeks (11.8% vs. 16.8%, respectively; *p* = .209) and 1 month (10.5% vs. 14.2%, respectively; *p* = .319) postpartum. For the secondary outcome, The NRS score of wound pain (3.0 ± 0.9 vs. 4.0 ± 1.0, respectively; *p* < .001) and uterine contraction pain (3.0 ± 0.9 vs. 4.1 ± 0.9, respectively; *p* < .001) was lower in the ketamine group at 2 days postpartum compared with placebo group. The sleep duration at 2 days did not differ between groups (4.8 ± 2.3 vs. 4.4 ± 2.0, respectively; *p* = .115) (Table 2). The incidence of headache was higher in the ketamine group at 5 min after dosing and that of hallucinations and dizziness were higher at both 5 and 15 min after dosing (Table 3).

**TABLE 2:** Outcome measures of subject

	Ketamine (*n* = 153)	Saline (*n* = 155)	*P*
2 days postpartum			
NRS score			
Wound pain	3.0 ± 0.9	4.0 ± 1.0	<.001
Contraction pain	3.0 ± 0.9	4.1 ± 0.9	<.001
Sleep duration	4.8 ± 2.0.3	4.4 ± 2.0	.115
1 week postpartum			
EPDS score	7.5 ± 2.2	8.2 ± 2.0	<.001
Occurrence of postpartum depression	20 (13.1%)	35 (22.6%)	.029
2 weeks postpartum			
EPDS score	7.4 ± 2.0	7.8 ± 2.1	.155
Occurrence of postpartum depression	18 (11.8%)	26 (16.8%)	.209
1 month postpartum			
EPDS score	7.3 ± 2.1	7.5 ± 2.2	.366
Occurrence of postpartum depression	16 (10.5%)	22 (14.2%)	.319

**TABLE 3:** Side effects after administration during the operation

	Ketamine (*n* = 153)	Saline (*n* = 155)	*p*
Vomiting 5 min	4 (2.6%)	2 (1.3%)	.668
15 min	3 (2.0%)	1 (0.6%)	.606
Out of operation room	1 (0.7%)	0 (0%)	.497
Headache 5 min	15 (9.8%)	3 (1.9%)	.003
15 min	6 (3.9%)	1 (0.6%)	.122
Out of operation room	2 (1.3%)	1 (0.6%)	.991
Hallucination 5 min	24 (15.7%)	0 (0%)	<.001
15 min	6 (3.9%)	0 (0%)	.014
Out of operation room	1 (0.7%)	0 (0%)	.497
Dizziness 5 min	56 (36.6%)	0 (0%)	<.001
15 min	34 (22.2%)	2 (1.3%)	<.001
Out of operation room	2 (1.3%)	1 (0.6%)	.991

## 4 DISCUSSION

The important finding of this study was that operative intravenous ketamine (0.25 mg/kg) could reduce the depressive symptoms for 1 week.

The rate of PPD is higher among developing countries like China. What's more, as the implementation of the second child policy, the number of elderly women attempting to bear children has increased. The quality of woman's eggs and a man's sperm declined dramatically with increasing age, leading to an increased risk of pregnancy‐related complications and rate of cesarean delivery among older women, which may lead to the increased incidence of PPD (Li & Deng, 2017). They may have some of the following concerns related to their pregnancy: the loss of some occupation‐promotion, opportunities, worries about the risk of health problems, during pregnancy and the neonatal period, the pressure of social and economic factors, and depression and other negative emotions that may increase the incidence of postpartum depression. PPD is also a strong predictor of suicidality in the postpartum period (Do, Hu, Otto, & Rohrbeck, 2013). The risk factors of suicidal ideation of new mothers are higher self‐esteem, lower social support and severe depression. Moreover, suicidal ideation after delivery is significantly correlated with thoughts of hurting baby (Babu, Subbakrishna, & Chandra, 2008). As a result, it is important to screen PPD and treat it at an early time.

In recent years, ketamine has been found to produce rapid amelioration of major depressive disorder (Han et al., 2016). A study reported that ketamine anesthesia provided faster response and remission after electroconvulsive therapy (ECT) compared with propofol anesthesia. However, there was limited evidence said ketamine could treat unipolar or bipolar depression (McCloud et al., 2015). More high‐quality RCTs are needed to determine the different efficacy of ketamine for unipolar depression and bipolar depression. In psychiatry, the existing antidepressant medications show limited effectiveness and delayed clinical response (I). So, it is of much interest to see whether the rapid efficacy of ketamine can bring more benefits to patients. Studies found ketamine could rapidly (within 1 day) and significantly reduced suicidal ideation and its effect continued for up to 1 week (Wilkinson et al., 2018). Adjunctive ketamine demonstrated a greater reduction in clinically significant suicidal ideation in depressed patients within 24 hr compared with midazolam, partially independently of antidepressant effect (Grunebaum et al., 2018). The rapid efficacy of ketamine can make up for the delayed clinical response of traditional antidepressants.

Ketamine had also been explored for improving postoperative depressive state. In depressed patients undergoing surgeries, small‐dose ketamine improved the postoperative depressive state and relieved postoperative pain (Kudoh, Takahira, Katagai, & Takazawa, 2002). They believed patients with symptoms of depression have increased postoperative pain. As a result, pain relief might contribute to an improvement in the depressive state. The founding that Ketamine had a rapid antidepression effect raised the possibility intraoperative administration of ketamine might improve postoperative mood and enhance resilience in the setting of surgical stress. A randomized trial of healthy (ASA physical status 1 or 2) surgical patients receiving subanesthetic ketamine during induction of anesthesia found positive effects on postoperative depressive symptoms (Jiang et al., 2016). The intraoperative application of ketamine was associated with improved scores for depressed mood and increased serum brain‐derived neurotrophic factor levels. However, one study had a contrast conclusion. When using older adults who were undergoing major surgery, the administration of ketamine during prolonged general anesthesia created unfavorable neural conditions for antidepressant effects at both cortical and subcortical levels (Mashour et al., 2018). In our study, we used ketamine among “healthy” subjects who had not diagnosed as depression. As an anesthetic and analgesic drug, ketamine had been widely used for the prevention of postoperative pain (Porter et al., 2015). Another study found intravenous low‐dose ketamine (0.15 mg/kg) combined with intrathecal bupivacaine for cesarean delivery provides longer postoperative analgesia and lower postoperative analgesic consumption than bupivacaine alone (Sen, Ozmert, Aydin, Baran, & Caliskan, 2005). Instead of preventing depressive symptoms, using ketamine can provide better analgesia which is commonly used in the realm of anesthesia. That is the reason why we use it among “healthy” subjects.

We chose 1 week as the timing to evaluate the degree of postpartum depressive symptoms for it was the duration that ketamine could be effective. A randomized, double‐blind, clinical trial found single bolus low dose of ketamine did not prevent postpartum depression (Xu, Li, et al., 2017). No significant differences were found in the prevalence of postpartum depression at 3 days and 6 weeks after delivery. The timing when ketamine could provide the best efficiency is still remained to be seen. Postpartum depressive symptoms which was measured 1 week postpartum were neither too short to be influenced by the postoperative changes nor too long. When the time passed on, the influence of a single dose of ketamine was getting smaller. The other factors, like economic status and the employment status of the spouse, health problems in the newborn, problems with family and spouse, reduced social support, might play more important roles in the happening of postpartum depressive symptoms (Ozcan, Boyacioglu, & Dinc, 2017). Because of these social and economic confounders, the benefits of ketamine might be covered. That might be also the reason why we did not find any difference in the degree of postpartum depressive symptoms between two group at 2 weeks and 1 month postpartum.

However, the effect of ketamine during the short time postpartum still do much benefits to new mothers. As we mentioned before, PPD is a strong predictor of suicidality in the postpartum period (Do et al., 2013). Screening positive for depression at 1–2 days postpartum is significantly correlated with suicidal ideation after delivery (Bodnar‐Deren, Klipstein, Fersh, Shemesh, & Howell, 2016). What's more, compared with before delivery, there is a trend of lower depression level, but higher suicidal ideation at early postpartum stage. As a result, ketamine used at an early time postpartum might can reduce the suicidal rates of new mothers with serious PPD.

A huge number of clinical trials explored the use of ketamine for the management and prevention of postoperative pain (Elia & Tramer, 2005). It was believed that low‐dose ketamine (0.1–0.5 mg/kg) might relieve postoperative pain management when used as an adjunct to local anesthetic, opioids, or other analgesic agents (Kohrs & Durieux, 1998; Schmid, Sandler, & Katz, 1999). We observed that postoperative analgesic consumption was significantly lower in ketamine group at 2 days after delivery, compared to saline group. The finding was similar to some previous studies (Kudoh et al., 2002; Nitta, Goyagi, & Nishikawa, 2013). Another study found no additional postoperative analgesic benefit of low‐dose ketamine during cesarean delivery in patients who received intrathecal morphine and intravenous ketorolac, but the subjects who received ketamine reported lower pain scores 2 weeks postpartum (Bauchat et al., 2011). They found no evidence of the relationship between ketamine and analgesic efficacy in a near time after operate. However, they indicated that ketamine might relieve chronic pain. What made the differences may because we just used loxoprofen sodium tablets for breakthrough pain without continuous analgesia, making the small analgesic benefit of one dose ketamine easier to be observed.

In our study, we observed a higher incidence of hallucination and dizziness in the ketamine group, but it did not last for long time. The most common acute psychiatric side effect of ketamine is anxiety; the others include agitation or irritability, euphoria or mood elevation, delusions or unusual thoughts, panic, and apathy. The most common psychotomimetic side effect reported was dissociation, followed by perceptual disturbance, odd or abnormal sensation, derealization, hallucinations, feeling strange, weird, bizarre, or unreal, and depersonalization. No long‐term psychotomimetic side effects were reported. However, those short‐term effects still reminded us of the safety when we use ketamine. It was necessary to recognized the major gaps that remain in our knowledge about the safety of ketamine. Future research is needed to address these unanswered questions and concerns (Sanacora et al., 2017).

There are several limitations in our study. First, we did not test the score of EPDS before delivery. PPD can also happen before the birth of child. From previous studies, ketamine might improve mood better in patients who are depressed than patients who are not. It will be more specific if we use ketamine in patients who have PPD and make it easier to see whether ketamine can help to improve the state of PPD. Second, besides the personal information of the subjects we recorded, like the perinatal complications and depression before, the social and family factors including low social support, marital difficulties, and negative life events can raise the risk of getting PPD, as well. What's more, the unwanted pregnancy or unwanted infant sex can also make an influence (Shitu, Geda, & Dheresa, 2019). We studied the employment of the couples which has no differences between groups, but failed to compare other factors among our groups, which might lead to some unknown bias. Third, we excluded the subjects with diagnosed depression before, who are considered as at higher risks of suffering from PPD. Because there would be more psychiatric confounders among that people than those who had never experienced depression before. Considering the limited sample size of our study, we excluded them. However, it might be of greater clinical significance to study the prevention of PPD among these people. Further related researchers may be done in the future. Fourth, although we recommended the subjects whose EPDS scores were higher than threshold to go to the psychiatric clinic to ascertain the diagnoses of PPD, we failed to continue follow‐up. As a result, we did not know the prevalence of PPD between groups.

In conclusion, we found a subanesthetic dose of ketamine (0.25 mg/kg) administered in cesarean delivery can reduce postpartum depressive symptoms at 1 week postpartum. We were not sure about the antidepressant effect of ketamine on woman diagnosed as PPD. Studies are needed to assess the safety and the efficacy of ketamine in women with high risks of getting PPD and its antidepressant effect on women have PPD.

## CONFLICT OF INTEREST

None declared.

## AUTHOR CONTRIBUTIONS

Yao J: Carried out the study and wrote the manuscript. Song T: Helped patient recruitment and data collection. Zhang Y and Guo N: Helped data collection. Zhao P: Designed and supported this study.

## Data Availability

All data generated or analyzed during this study are included in this article.
